# The climate crisis and forensic mental healthcare: what are we doing?

**DOI:** 10.1192/bjb.2020.36

**Published:** 2021-02

**Authors:** Jack Tomlin

**Affiliations:** Department of Forensic Psychiatry, University of Rostock, Germany

**Keywords:** Forensic mental health services, climate crisis, sustainability, climate action, sustainable mental health

## Abstract

The climate crisis poses the greatest threat to human health this century. Mental health services will be called on to address the psychological consequences of its effects on peoples’ lives, particularly the socially disadvantaged and those on low incomes. However, healthcare systems are also contributors to the climate crisis. This editorial discusses how services can continue to provide care while contributing less to climate change. Specifically, it suggests what services such as forensic mental healthcare, which is constrained by legal, political and resourcing concerns, can do differently.

Ten years ago, *The Lancet* identified climate change as the largest threat to human health in the 21st century.^1^ Changes in the environment and biodiversity, alongside unpredictable natural disasters, will have consequences for both the somatic and mental health of the world's population. Individual and collective mental health are likely to suffer owing to anxiety over fluctuating living conditions, loss of means of income, broken social bonds and conflict linked to resource scarcity, with low income and socially disadvantaged groups most likely to be affected.^2–4^

Mental health systems will play a significant role in responding to the psychological fallout of the climate crisis. Services will have to respond to experiences of trauma following natural disasters and longer-term mental health concerns, such as depression and anxiety, linked to changes in lifestyles, environmental damage and resource scarcity.^3^ A study conducted 1 year after the 2005 Hurricane Katrina in New Orleans, USA, found that 40% of 144 surveyed residents had a probable mental illness, half of which were classified as severe. This contrasted with a survey conducted between 2001 and 2003, which estimated that 16% of respondents in the region had any type of mental illness.^5^ The authors attributed this to destroyed or damaged housing and property, consequent dislocation and associated losses of employment and community ties.

## The healthcare sector's contribution to the problem

Most causes of climate change are well acknowledged: overreliance on fossil fuels, poisonous greenhouse gas emissions, unsustainable agricultural practices. Yet paradoxically, while being tasked with managing much of the future damage to human well-being, healthcare systems are themselves contributors to the climate crisis. In the USA, a ‘top emitter’, 7.6% of national emissions (defined as carbon dioxide, methane and nitrous oxide) come from the healthcare sector (1.72 tCO_2_e/capita).^6^ This is the equivalent to 141 coal power plants. In the UK, the healthcare system contributes 5.4% (0.66 tCO_2_e/capita); the average across the European Union is slightly lower: 4.7% (0.49 tCO_2_e/capita). A ‘lower than average emitter’, India's healthcare system contributes 1.5% to total emissions (58 times less than the USA per capita).

These contributions derive from the manufacture of pharmaceutical products; the large amounts of resources needed to run equipment, heat buildings and transport patients or staff to appointments; and the disposal of waste products.^7^ As health and social care services constitute a substantial part of every country's economic and social activity, the magnitude of their contribution may be understandable. However, given the preventive and life-saving functions that healthcare systems provide, significant questions remain – how can healthcare systems provide perennially improving, high-quality care while contributing less to the climate crisis? And what does this mean for sectors particularly constrained by legal, political and resourcing concerns, such as forensic mental health services – especially when the number of forensic in-patient beds has been increasing in many North American and European states?^8^

## ‘Sustainability’ in mental healthcare: the ideal…

**Fig. 1:**
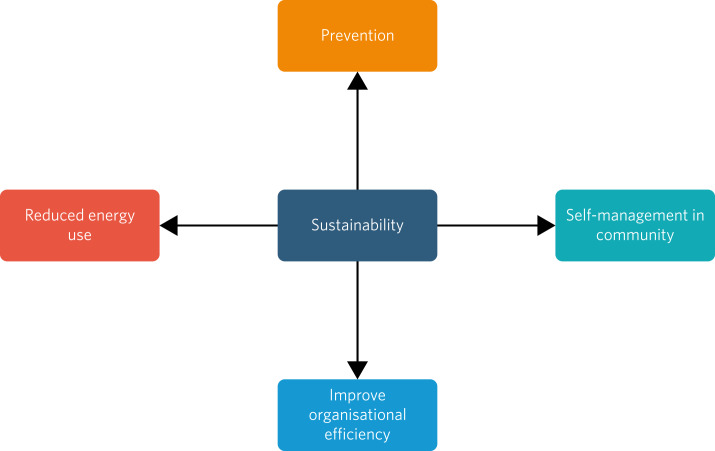
Four basic principles for sustainable mental healthcare.

Recent efforts have attempted to embed the concept of ‘sustainability’ into routine mental healthcare. Sustainable mental health services are those that weave sustainable thinking into decision-making at all levels. The Joint Commissioning Panel for Mental Health and the Centre for Sustainable Healthcare^7^ identify four basic principles that settings should adhere to. Services need to (a) promote patient self-management, (b) prioritise prevention instead of response to illness, (c) adopt sustainable methods of energy use and (d) increase efficiency in service provision (Fig. 1). These principles should not be understood as detracting from the necessity for gold-standard clinical care for those who need acute, urgent treatment to manage severe mental illness. This reactive medicine must still be available but with practitioners cognisant of a sustainable approach and alert to the notion that embracing these principles can help lower or buffer the growing future need for such care.

What do these principles look like in practice? Taken together, these recommendations aim to develop sustainable resource use. The more mental illness is prevented and managed in the community, the less secondary care services will be called on. When services are engaged, they should minimise and embrace clean-energy use and maximise efficiency, so that time, resources and medications are not wasted. Specific aims include avoiding hospital admissions that might have been identified earlier and managed in primary care; the development and monitoring of targets to reduce carbon emissions, including seeking more efficient methods of heating buildings and transporting food, medicines and materials, and reducing amounts of waste; enabling patients to self-manage symptoms in the community where possible; offering horticultural therapies, walking groups and psychological interventions instead of prescribing unnecessary medication; offering telephone or online interventions; and reducing the number of missed appointments.^9^

## …and the reality in forensic settings

However, the transferability of these aims to forensic settings is not immediately apparent. Forensic services are high cost and provide care to patients who are, in law and fact, deprived of their liberty. Patients are placed in care following the commission of a crime or when risk of harm is too difficult to manage in general or out-patient services. Thus, forensic settings reflect failures elsewhere to prevent or manage mental illness and antisocial behaviour. They require resource-intensive security measures; services may be reticent or unable to promote patient self-management owing to seriousness of illness or the custodial attitudes of staff and policy makers; patients may be kept within inappropriately high levels of security because of political or media attention; and they typically rely less on volunteers and carers from the local community to help manage patient recovery and run services than other health services might. Accordingly, efforts to meet the aims of prevention, self-management and reducing carbon emissions could face extra barriers.

## Some suggested steps forward

So, what can forensic services do? All services should develop a sustainable development plan. Such a plan details aims, objectives, strategies and priorities for improving local environmental and socioeconomic impacts and should set measurable targets.^10^ These should reference national or regional standards for reducing carbon emissions. These plans are already required by healthcare providers commissioned by standard contracts in the National Health Service in England and Wales. Patients should be involved in developing targets and action plans. This should be complemented by routine monitoring of procedural and substantive outcomes, including, for instance, whether sustainability is incorporated within a service's mission statement or the ways in which it is included in decision-making structures, reductions in waste and energy use, or the number of meals produced using food grown on-site. Plans can draw on the four basic principles described earlier (Fig. 1).

Steps should be taken to integrate patients into local communities to promote self-management of symptoms, prevent mental health problems or risky behaviour due to social isolation or deskilling, and thus reduce need for services. Peer support programmes run by former forensic services patients or carers, work for patients outside secure settings, and proactive attitudes towards granting leave could all be helpful. Treatment paradigms such as the recovery approach and the Good Lives Model that aim to improve the quality of patients’ lives holistically, promote recovery and target criminogenic factors as well as treating mental disorders should be used to increase the chance of successful rehabilitation that carries though into the community and reduces future service use.^11,12^

Punitive attitudes that discourage the placement of patients in lower levels of security closer to the community should be tackled by implementing training programmes that educate all staff on the antecedents, symptoms and prognoses of patient diagnoses and thus encourage a therapeutic mindset. The unnecessary use of medications should be avoided. When tension or aggression is present on a ward then the use of verbal de-escalation techniques by appropriately trained staff might avoid the requirement for ‘as needed’ (p.r.n.) medications.^13^

Finally, individuals working in secure services should develop a network to (a) share sustainable development plans and best practices, (b) identify challenges unique to forensic settings and (c) connect with individuals in general mental health and somatic care who have already developed such networks (e.g the Centre for Sustainable Healthcare in the UK). Forensic services should be aware of how they contribute to and can help alleviate the consequences of the greatest threat to human health in the 21st century. To avoid doing so would be to ignore our guiding principles of reducing harm and improving lives.

## About the author

**Jack Tomlin** is a Research Fellow in the Department of Forensic Psychiatry, University of Rostock, Germany.

## Supplementary material

For supplementary material accompanying this paper visit https://doi.org/10.1192/bjb.2020.36. click here to view supplementary material
